# Empowering Deaf-Hearing Communication: Exploring Synergies between Predictive and Generative AI-Based Strategies towards (*Portuguese*) Sign Language Interpretation

**DOI:** 10.3390/jimaging9110235

**Published:** 2023-10-25

**Authors:** Telmo Adão, João Oliveira, Somayeh Shahrabadi, Hugo Jesus, Marco Fernandes, Ângelo Costa, Vânia Ferreira, Martinho Fradeira Gonçalves, Miguel A. Guevara Lopéz, Emanuel Peres, Luís Gonzaga Magalhães

**Affiliations:** 1 Department of Engineering, School of Sciences and Technology, University of Trás-os-Montes e Alto Douro, 5000-801 Vila Real, Portugal; eperes@utad.pt; 2 ALGORITMI Research Centre/LASI, University of Minho, 4800-058 Guimarães, Portugal; miguel.lopez@estsetubal.ips.pt (M.A.G.L.); lmagalhaes@dsi.uminho.pt (L.G.M.); 3 Centro de Computação Gráfica-CCG/zgdv, University of Minho, Campus de Azurém, Edifício 14, 4800-058 Guimarães, Portugal; joaooliveira@ccg.pt (J.O.); somayeh.shahrabadi@ccg.pt (S.S.); jose.jesus@ccg.pt (H.J.); 4 Polytechnic Institute of Bragança, School of Communication, Administration and Tourism, Campus do Cruzeiro, 5370-202 Mirandela, Portugal; a35106@alunos.ipb.pt (M.F.); martinho.goncalves@ipb.pt (M.F.G.); 5 Associação Portuguesa de Surdos (APS), 1600-796 Lisboa, Portugal; angelomscosta@gmail.com (Â.C.); vania.ferreira.91@hotmail.com (V.F.); 6 Instituto Politécnico de Setúbal, Escola Superior de Tecnologia de Setúbal, 2914-508 Setúbal, Portugal; 7 Centre for the Research and Technology of Agro-Environmental and Biological Sciences, University of Trás-os-Montes e Alto Douro, 5000-801 Vila Real, Portugal; 8 Institute for Innovation, Capacity Building and Sustainability of Agri-Food Production, University of Trás-os-Montes e Alto Douro, 5000-801 Vila Real, Portugal

**Keywords:** sign language recognition (SLR), Portuguese Sign Language, video-based motion analytics, machine learning (ML), long-short term memory (LSTM), large language models (LLM), generative pre-trained transformer (GPT), deaf-hearing communication, inclusion

## Abstract

Communication between Deaf and hearing individuals remains a persistent challenge requiring attention to foster inclusivity. Despite notable efforts in the development of digital solutions for sign language recognition (SLR), several issues persist, such as cross-platform interoperability and strategies for tokenizing signs to enable continuous conversations and coherent sentence construction. To address such issues, this paper proposes a non-invasive Portuguese Sign Language (*Língua Gestual Portuguesa* or LGP) interpretation system-as-a-service, leveraging skeletal posture sequence inference powered by long-short term memory (LSTM) architectures. To address the scarcity of examples during machine learning (ML) model training, dataset augmentation strategies are explored. Additionally, a buffer-based interaction technique is introduced to facilitate LGP terms tokenization. This technique provides real-time feedback to users, allowing them to gauge the time remaining to complete a sign, which aids in the construction of grammatically coherent sentences based on inferred terms/words. To support human-like conditioning rules for interpretation, a large language model (LLM) service is integrated. Experiments reveal that LSTM-based neural networks, trained with 50 LGP terms and subjected to data augmentation, achieved accuracy levels ranging from 80% to 95.6%. Users unanimously reported a high level of intuition when using the buffer-based interaction strategy for terms/words tokenization. Furthermore, tests with an LLM—specifically ChatGPT—demonstrated promising semantic correlation rates in generated sentences, comparable to expected sentences.

## 1. Introduction

Ensuring full participation of individuals in every aspect of life, regardless of background, gender, culture, age, or condition is one of the great goals of inclusion. More particularly, in the case of Deaf persons, it is of utmost importance to adapt environments and develop interaction models for bridging them with the rest of society, enabling suitable integration where the lack of auditory sense is no longer an issue. The Convention for Portuguese Sign Language (*Língua Gestual Portuguesa* or LGP), established in 1823 and with origins associated with Casa Pia, Lisboa [1], has evolved over time, combining gestures involving hand configurations, movements, facial expressions, and body postures for communication. While LGP is widely used within the Deaf Portuguese community, it remains unfamiliar to individuals with auditory capabilities, emphasizing the need for strategies to promote better inclusion.

In this context, technology offers promising solutions, as shown in the prequel of this work [2], focused on acquiring LGP videos, constructing datasets, and conducting preliminary tests using long-short term memory (LSTM)-based approaches, wherein an 86% accuracy rate in image-based gesture/sign recognition was achieved. While similar works in the literature share the goal of sign language recognition (SLR), it is crucial to distinguish between static and moving signs. Static signs involve hand and body poses that remain stationary throughout the action, while moving signs incorporate gesture-based procedures with changing hand and body positions over time, until the accomplishment of a required expression. While [3] has classified video frame-by-frame to obtain the highest probability associated with a sign, in practice it disregards the motion analytics underlying video sequences. Various approaches exist using invasive devices, such as sensor gloves [4] or non-mainstream technology such as RGB-Depth (RGB-D) sensors [5]. Others, which yielded promising results, focus on sequence analysis, utilizing LSTM networks [4], 3D convolution with LSTM networks [6], or transformer models [7]. Approaches such as [8] have focused on hand features, particularly landmarks, achieving high accuracy with extensive datasets. To address continuous SLR, [9] proposed a multi-information spatial-temporal LSTM (ST-LSTM) fusion technology, processing videos of uninterrupted sign communication and making text-based inferences. Despite progress in this field, the development of SLR-as-a-service remains under-explored in the literature, with few publicly available solutions (e.g., [10]). As for computer-based pattern mapping centered on training inference models, there is a wide set of publicly available datasets (e.g., contributions [11,12]) targeting distinct sign languages that vary from country to country, and which can be categorized into single-word and multiple-word collections.

Furthermore, with recent groundbreaking advancements in natural language generation, particularly with the development of large pre-trained language models (LLMs) such as Bidirectional Encoder Representations from Transformers (BERT) [13] and ChatGPT (v.4) [14], the conditioned transformation of clouds of vague, although, semantically relatable concepts—such as those resulting from sign recognition tasks—into grammatically coherent sentences is a tangible reality, widely disseminated and available to anyone with an internet connection and a web browser access.

In light of the aforementioned challenges and opportunities, this paper documents the research and implementation activities carried out to propose a novel sign language interpretation approach for LGP supported by both generative and predictive AI-based strategies, with the following fine-grained goals in mind: (a) to develop a web-based LGP recognition solution, as-a-service; (b) to propose a fully original 50 sign dataset composed of dynamic LGP sings; (c) to explore different data augmentation strategies aiming to improve inference models learning skills; (d) to compare LSTM variants for textual term inference from video-based signs; (e) to propose a frame buffer-based human-computer interaction (HCI) technique for word segmentation; and (f) to harness LLM (ChatGPT) generation capabilities to build semantically meaningful sentences, conditioned by rules.

The paper is organized as follows: the second section presents related work, while the third section details the system proposal. The fourth section covers implementation, the fifth section presents tests and results, and the sixth and final section summarizes the work and outlines potential future research paths.

## 2. Related Work

In the domain of SLR, several noteworthy approaches and technologies have emerged in recent years. These efforts aim to bridge the communication gap between Deaf and hearing individuals while addressing the challenges posed by the diversity of sign languages and the intricacies of interpreting gestures.

Many works in the literature focus on recognizing sign language through different modalities, including image-based recognition, and sensor-based approaches, most of them resorting to machine or, even, deep learning techniques to perform word/term recognition/association. Regarding body motion dynamics, some studies have explored the recognition of static signs using image-based techniques. For example, in [5], the authors concentrate on alphabet letters and numbers, primarily static gestures. While these approaches can be simpler than dealing with motion analytics, they may not capture the nuances of dynamic signs and can be sensitive to environmental conditions, as highlighted in [15].

Some works require the use of specific devices for SLR. In [4], signs were collected through a sensor glove, providing precise data but involving an invasive approach. Alternatively, in [5], a Microsoft Kinect with RGB-Depth (RGB-D) sensors was utilized, which, while effective, may not represent a mainstream technology, at least, comparatively to the popular RGB webcams that can be commonly found integrated with laptops or smartphones.

Recognizing the sequence of signs, especially the dynamic ones, has gained attention in recent research. Approaches involving LSTM networks [4] or combining 3D convolution with LSTM networks [6] have demonstrated high accuracy. Transformers have also been applied to SLR tasks, as seen in [7], where body landmarks and pose vectors were used to achieve good performances, as well.

The development of publicly available sign language datasets has been essential for training and evaluating SLR systems. For example, the Indian Lexicon Sign Language Dataset (INCLUDE) [16], the Word-Level American Sign Language (WLASL) [11], the Argentinian Sign Language with 64 examples (LSA64) [12], the RWTH-PHOENIX-Weather2014T [17], the How2Sign [18], and the *Língua Brasileira de Sinais* from Universidade Federal de Ouro Preto (LIBRAS-UFOP) [19] have provided valuable resources for researchers.

Despite these advancements, the concept of SLR-as-service remains relatively unexplored in the literature. Few publicly available solutions have been developed, such as SLAIT [10] and PopSignAI [20], which focus, respectively, on sign interpretation assistance and teaching, both encompassing American Sign Language (ASL). However, since these solutions serve commercial purposes, there is no technical or scientific information openly accessible to deepen the knowledge regarding the research and implementation carried out to support the respective applications. Consequently, issues intrinsically associated with architectures capable of providing PSL as-a-service, including concerns that extend to data formats and communication strategies for tackling bandwidth limitations and, therefore, ensuring (near) real-time operationalization, still require further study and exploration.

Furthermore, the integration of large pre-trained language models (LLMs), such as BERT [13] or ChatGPT [14], offers exciting possibilities for generating grammatically coherent sentences based on sign language inputs. These models have shown impressive abilities in capturing and leveraging linguistic-aware capabilities, which may be explored through prompting approaches that allow to regulate outputs, and to customize responses in tasks ranging from text generation to translation, as well as summarization, unleashing new frontiers for natural language applications.

Recent studies have introduced innovative prompting techniques, showcasing the versatility of LLMs in various language tasks. For instance, [21] presents methodical prompting that enhances linguistic structure capture through sequence tagging using GPT-3. This approach aims to evaluate whether the language skills of LLMs are primarily linked to generalizable linguistic understanding or surface-level lexical patterns. In [22], a systematic approach using the Socratic method to develop prompt templates was proposed, leading to improved interaction with LLMs. For example, while definition, elenchus, and dialectic methods are suitable to clarify user queries, maieutics, and counterfactual reasoning are more helpful to stimulate story writers’ creativity. Another work [23] conducted a case study on prompting for machine translation using GPT-3, demonstrating its effectiveness in enhancing translation quality. Furthermore, in [24], an approach to building chatbots was proposed by leveraging LLMs and accepting natural language prompts, contributing to more accurate and contextually relevant natural language processing applications.

In summary, while significant progress has been made in SLR and the integration of LLMs, there remains room for exploration in SLR-as-service and the development of coherent sentence-generation models.

## 3. LGP-to-Text System Specification

In the context of developing digital solutions that are grammar-sensitive and aligned with the LGP structure, crucial knowledge resources are available for reference, including [25]. Additionally, Portuguese organizations such as the *Associação Portuguesa de Surdos* (APS) offer valuable insights into the LGP grammatical structure. Specific rules that pertain to this work can also be found in a previous contribution [2]. With this background in mind, Section 3 is dedicated to presenting the concept and specification of the proposed Deaf-hearing communication system.

### 3.1. Main Architecture

**Figure 1:**
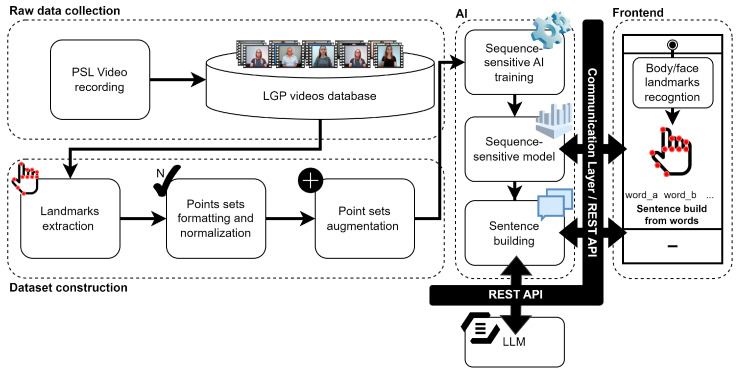
Main architecture for the proposed Deaf-hearing communication system, composed of four modules: (i) raw data collection consisting of the recording of labeled videos; (ii) dataset construction for structuring the raw data into an AI-complaint organization; (iii) AI consortium of logical entities responsible for inferring words/terms out of LGP signs-based anatomical landmarks, and for outputting well-structured sentences, in a rule-based conditioned manner by resorting to an LLM; and (iv) frontend, for interfacing with the Deaf individuals.

The main architecture of the SLR solution, illustrated in Figure 1, comprises four essential modules: (i) raw data collection; (ii) dataset construction; (iii) AI; and (iv) frontend. The raw data collection module encompasses the processes for recording and storing labeled sign language videos contributed by users. As for the dataset construction, it involves a series of subsequent operations on the labeled raw videos, including:

- Extraction of relevant features, namely anatomical landmarks points sets;
- Normalization of these points sets into a structure compatible with AI processing;
- Application of augmentations to both video frames and extracted point sets.

The AI module is implemented as a service accessible through Representational State Transfer (REST)-based Application Programming Interface (API). Finally, the frontend module provides a user-friendly interface for Deaf individuals to interact with the system, ensuring seamless compatibility with the REST API methods. These interoperable capabilities allow external parties to use the proposed SLR-system-as-a-service, offering flexibility and scalability.

### 3.2. Acquisition Campaign and Steps for Structuring Data

Due to the scarcity of publicly available LGP datasets, especially those involving language signs procedures’ motion dynamics, a data collection campaign based on video recordings was carried out. Central to this effort was the meticulous labeling of each recorded video, a crucial step in this supervised approach to performing SLR. To ensure the fidelity of the data, LGP experts were engaged as contributors. These experts, including LGP interpreters, LGP teachers, and Deaf individuals who rely on well-formed LGP for daily communication, were instrumental in capturing precise body, hands, arms, and facial postures, as well as motion-specific features and sequences. As part of this collaborative effort, several APS associates were included to perform a great part of the recordings of the signs that ultimately formed the basis of the final datasets. To guide their contributions, the following instructions were provided:

- Contributors should position themselves at the center of the camera’s frame, ensuring roughly the same space at both lateral margins (left and right);
- Their background should be kept free of other people or visible elements that could resemble people, besides themselves;
- Gestures should be made calmly and slowly, allowing the camera to capture most of the motion details;
- The place chosen for acquiring the videos should be uniformly illuminated, avoiding excessive darkness while also preventing overexposure caused by strong and direct source lights;
- Videos should be labeled in agreement with the recorded signs.

To aid in increasing the number of labeled examples, non-expert contributors were also requested to faithfully reproduce LGP signs, by assisting, analyzing, and trying to imitate the sequences expressed in the videos already recorded by the LGP experts, while following the same instructions. For signs that lacked examples to follow, non-experts were instructed to refer to an official source, namely the online LGP dictionary of Infopedia [25].

With the aim of achieving a sufficient lexical range to shape up an adequate variety of sentences in the context of commerce, tourism, and health, a list of 50 Portuguese terms/words has been specified for the data collection campaign. Table 1 presents that list, which includes the respective English translations.

Table 2 summarizes the key statistics related to the data collection campaign and the initial data structuring process, which served as the basis for subsequent preprocessing steps, leading to the final dataset assembly. A total of 33 examples were recorded for each of the 50 specified signs. Subsequently, it was determined that, for each sign, 28 examples would be randomly selected for training the AI models, while the remaining five would be used for evaluating inference capabilities. Within the training set, ≈65% was established as the reference rate for the models’ training process, leaving the remaining ≈35% allocated for validation purposes.

### 3.3. Strategy for Data Preparation: From Augmentation to Dataset Arrangement

**Figure 2:**
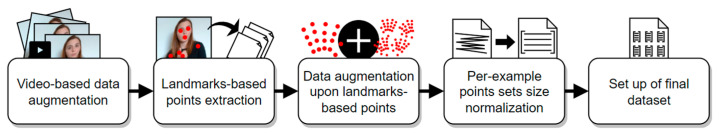
Pipeline for data preparation, including a few data augmentation strategies (video- and point-based), as well as normalization steps, until the consolidation of the final dataset.

After dividing data, several additional steps are necessary (Figure 2), including video-based augmentation, anatomical landmarks-based points extraction, per-example points set size normalization, and data augmentation upon landmarks-based points. These steps lead to the formation of the final dataset.

To enhance data diversity through video-based augmentation, various transformations can be applied to simulate different conditions, particularly focusing on the sensor (camera) capturing perspectives. For instance, introducing distortions to the video frames can generate synthesized and valuable data.

When it comes to landmark-based point extraction it is essential for capturing key anatomical landmarks from each frame to characterize signs based on their motion and spatial attributes. During this extraction step, it is beneficial to crop frames in a way that preserves the visual information of the contributor’s body, with their head approximately aligned with the center of the horizontal axis. These procedures contribute not only to favor data normalization (e.g., by keeping contributors at the center of the frames) but also to work with filtered data, eventually more resilient to environmental conditions (e.g., by reducing contributors’ background). Additionally, it is worth noting that points are only extracted when at least one of the hands is detected. Frames that do not allow the detection of such points are considered unusable and, therefore, are discarded.

**Figure 3:**
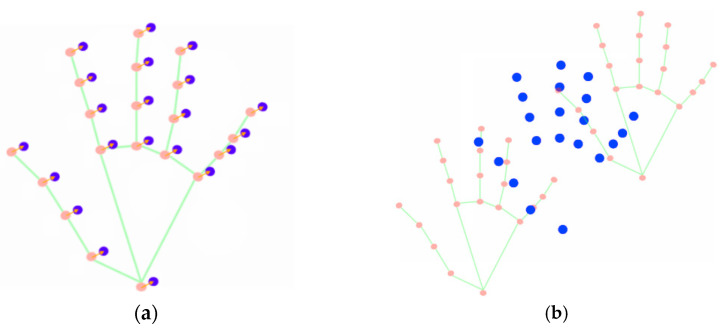
Data augmentation operations applied to original key points: (**a**) illustrates the RRS process, and (**b**) depicts the SBI process. In both cases, semi-transparent red dots represent actual anatomical key points (collected from contributors), while purple/blue dots represent augmented key points. For simplicity, the anatomical key points of a single hand are shown, for each presented data augmentation operation.

The next step in the pipeline focuses on data augmentation centered around landmarks-based points. This aims at enhancing the dataset quantity—by introducing synthesized variations—while retaining the inherent landmarks’ significance, which is more associated with data quality. One operation that contributes to consistency-oriented data augmentation is horizontal flips. These help mitigate bias that may be induced by contributors’ lateral dominance, meaning their proneness to perform signs according to their dominant side. Additionally, operations involving displacement noise, such as random radial shifts (RRS), and interpolations, specifically spline-based interpolation (SBI) are performed as depicted in Figure 3. RRS resorts to the normalized original positions of the skeletal-based landmarks and applies small-intensity displacement entropy to each anatomical group (hands, arms, face, and torso). On the other hand, SBI involves multiple passes to add intermediate coordinates in the middle of the existing ones, extending the data. After this extension, SBI creates N groups evenly populated with sequences of points, wherein N matches the number of expected point sets as defined by the cardinality of the normalized data, and, per group, selects randomly a point to be part of the final series.

The RRS operations are detailed in the pseudo-code shown in Algorithm 1, and the pseudo-code associated with the BSI approach is presented in Algorithm 2.
**Algorithm 1**: Pseudo-code responsible for skeletal landmarks-based key points RRSInput: *landmarks_groups*: a dictionary containing groups of *key points* (x,y) lists. *radius_range_norm*: a range of radii for performing RRS (default [0.01, 0.05])Output: *landmarks_rrs*: a dictionary containing groups of *key points* induced with RRS variation
Begin
  1.Initialize *landmarks_rrs* as a copy of *landmark_groups*.  2.For each group in *landmarks_rrs*:  a.Generate a random radius within *radius_range_perturb*.  b.Generate a random angle between 0 and 2π.  c.Calculate perturbation values for x and y coordinates:   i.*perturbation_x* = *radius* ∗ sin(*angle*)  ii.*perturbation_y* = *radius* ∗ cos(*angle*)  d.For each *key point* as *kp* in the current group of *landmarks_rrs*:   i.Perturb the x-coordinates of *kp* by adding *perturbation_x*.  ii.Perturb the y-coordinates of *kp* by adding *perturbation_y*.  3.Return trimmed *landmarks_rrs*.EndNotes: (1) *landmarks_groups* contains groups of *key points* that are normalized from 0..1; (2) the *radius_range_norm* must take into account the previous assumption, and adequate the perturbation range accordingly; (3) trimming the landmarks will prevent out (normalized) values to be out of range;

**Algorithm 2**: Pseudo-code responsible for skeletal landmarks-based key points SBIInput: *landmarks_groups*: a dictionary containing groups of *key points* (x,y) lists. *n_points*: limit of points to interpolate for each coordinate set. *groups_per_point*: limit of groups to split the *key points* augmented.Output: *landmarks_sbi*: a dictionary representing the interpolated and normalized sequence of key points and coordinates.
Begin
  1.Initialize *landmarks_sbi* as a copy of *landmarks_groups*.  2.For each group in *sbi _rrs*:  a.Set *filtered_interpolated_values* as an empty array.  b.Set *listValues* with the list of *key points* of the current group.  c.Set *interpolated_values* with the result of   *interpolate_spline_normalized(listValues, n_points × groups_per_point)*.  d.Split *interpolated_values* into evenly spaced groups *esg*, with a division   matching *groups_per_point*.  e.For each *esg*, select a random interpolated *key point* and   append it to *filtered_interpolated_values*.  f.Update the coordinates set of the current group with   *filtered_interpolated_values*.  3.Return trimmed *landmarks_rrs*.EndNotes: (1) *landmarks_groups* contains groups of *key points* that are normalized from 0..1; (2) *interpolate_spline_normalized* is a recursive function that performs spline interpolation in a sequence of *x,y* points until a given limit (in this case, *n_points × groups_per_point*);

Based on the assumption that the key points associated with hand/arm landmarks typically describe a curve trajectory, spline interpolation seems to be the proper mathematical technique to add intermediary points for SBI-based data augmentation. This technique approximates a curve that passes through a given set of points and estimates complementary data between them. In this work, two uni-variate approaches were used, considering the key point sets: one to estimate the intermediate points in the x component and another to do the same for the y component. One commonly used type of uni-variate spline is the cubic spline, whose formula in a specific interval [$x_{i}$, $x_{i+1}$] can be expressed as in Equation (1).

(1) $$S_{i}\left(x\right)=a_{i}+b_{i}\left(x-x_{i}\right)+c_{i}{\left(x-x_{i}\right)}^{2}+d{\left(x-x_{i}\right)}^{3}$$

where:

$S_{i}\left(x\right)$ is the cubic spline function in the interval [$x_{i}$, $x_{i+1}$].
$a_{i}$, $b_{i}$, $c_{i}$, and $d_{i}$ are coefficients specific to the i-th interval.
$x_{i}$ is the starting point of the interval.
x is the input value within the interval.

The next step involves normalizing the data in terms of size. Indeed, all the extracted sets of points are normalized for each sign example, aiming to create uniform groups of landmark-based positions. For each sign collected in the previous landmarks-based points extraction step, a fixed number of intermediate sets are considered to facilitate the normalization process. After completing all the steps, a data lake is constructed that aligns with the results of the previously mentioned steps. The main outcome is the creation of a comprehensive and balanced dataset that complies with ML techniques to be applied in subsequent stages.

### 3.4. ML Powered by LSTMs for LGP Recognition

In the context of LGP communication, sign language involves a spatio-temporal relationship, where the chaining of sequences is of upmost importance. LSTM stands out as a reliable strategy, as demonstrated in previous works such as [8], for ML tasks. LSTM is a type of recurrent neural network (RNN) architecture specifically designed to handle sequences and time-series data. Unlike traditional RNNs, which may struggle with capturing long-range dependencies in sequences, LSTM networks have a unique structure comprising memory cells, gates, and input/output connections. This unique design enables them to selectively remember and forget information over extended periods, making them well-suited for various tasks, including natural language processing, speech recognition, time-series prediction, and more.

### 3.5. Tokenization Strategy—A Conditioning Interaction-Based Approach

**Figure 4:**
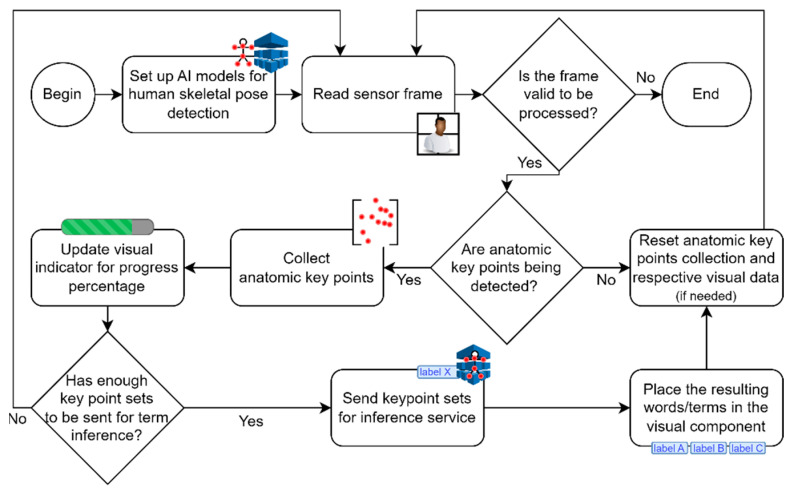
Flow diagram representing anatomic key points set buffer-based process, along LGP expression capturing.

To address one of the most significant challenges in LGP communication, which involves knowing the start and the end of a given term/word-related sign in a continuous conversation, an interaction technique is proposed. It is based on a visual representation of the filling progress of a (landmarks-based) key points set buffer. This approach conditions the user’s input and is depicted in Figure 4. Specifically, an ML-based detection approach to track the user’s skeleton is incorporated to recognize landmarks of the torso, arms, hands, and face, returning their respective *x,y* points. When at least one of the user’s hands is detected, points of the entire body, out of a predefined universe of 88 points, are collected into a buffer with a set limit. Any key points outside the frame range are assigned a value of −1 for both the *x* and *y* components. Simultaneously, visual feedback elements are provided to intuitively inform the user about the progress of the sign. The primary goal is to offer interactive guidance, enabling the user to synchronize each sign with the visual progress feedback, as a means of tokenizing the terms or words. When the key points set buffer reaches its limit, the points are sent to an LSTM-based inference service that returns the most statistically meaningful term or word from a previously learned set. In such cases, a reset of the key points set buffer is performed. Another method for resetting the buffer—causing it to discard all accumulated points without transmitting any data to the inference service—involves the use of a time counter process. This process accumulates milliseconds across consecutive frames in instances where the user’s hands are not detected by the skeleton detection model. Once a predefined threshold is reached, a reset to the key point set buffer occurs. This is particularly useful when the user wishes to cancel a sign that was started by mistake or went wrong during execution.

### 3.6. LLM-Based Sentence Construction

While interpreting LGP motion analytics is essential to extract words or terms that may provide guidance to the communication intentions of the LGP user, the resulting vocabulary often lacks grammatical structure, hindering the creation of meaningful sentences for clearer communication. Although natural language processing (NLP) commonly focuses on textual decomposition, analysis, and knowledge extraction [26]—it has been bringing significant developments to the machine-based understanding of content typically produced by humans, including in applications for translating European Portuguese to LGP-compliant gloss [27]—the challenge of constructing well-structured and meaningful sentences from word clouds requires generative approaches that go beyond typical NLP goals. Recent developments in computational syntax and semantics, particularly in LLMs have opened possibilities for natural language generation (NLG). These advancements enable AI agents to autonomously produce coherent conversations and interpret user-defined rules to provide contextually relevant outputs. Whether using traditional n-gram models or more advanced neural network-based models such as GPT, LMMs have the capability to assemble loose terms and words into human-like textual excerpts, ensuring contextual coherence along multiple iterations, if needed.

**Figure 5:**
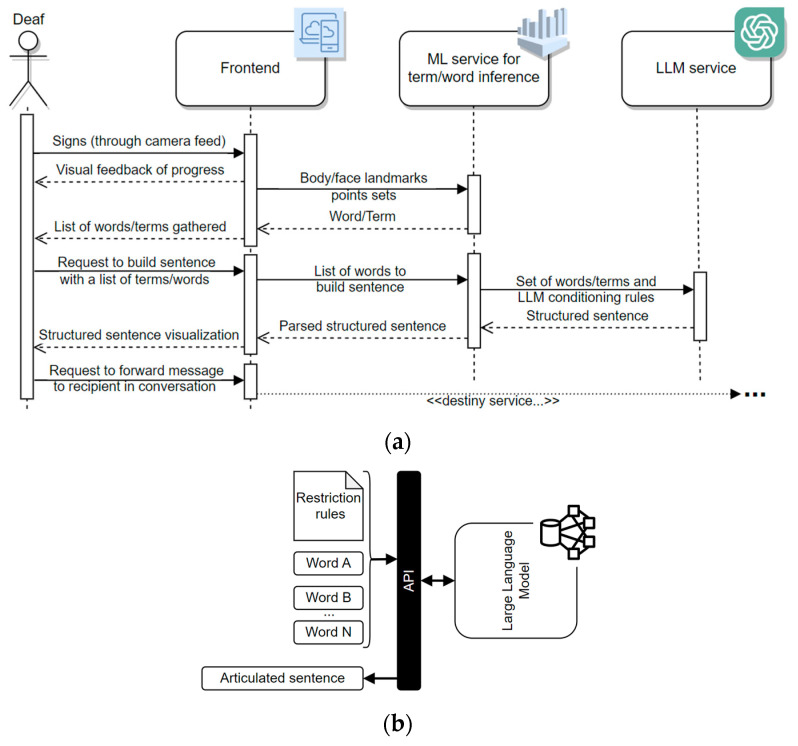
Communication sequence for LGP interpretation and sentence construction: (**a**) depicts the interaction of the user with the proposed LGP system and LLM service, through a frontend interface; (**b**) illustrates the interaction between LLM and the service requesters, involving a set of words and conditioning rules, resulting in the generation of coherent sentences.

In the context of LGP communication, LLM tools, such as those supporting GPT-3.5 [28], can be employed to construct sentences from LGP gloss. These models can also be fine-tuned with human-readable parameters to optimize their output, with human regulators playing a vital role in providing guidelines to enhance LLM responses, as needed. This work integrates LLM in the proposed LGP interpretation pipeline, as depicted in Figure 5. This diagram illustrates end-to-end interactions between an LGP user, a (detachable) frontend layer, an ML service for inferring terms and words based on user motion analytics, and an LLM service that constructs sentences using a vocabulary set as input. The process begins with the Deaf user providing LGP expressions to the frontend, captured through a camera sensor. Landmarks based on facial features, arms, hands, and torso are acquired up to a certain limit, which is visually indicated to the user. When the sign ends, these landmarks are sent to the ML service, which performs inferences and returns corresponding words or terms. After assembling a satisfactory set of words or terms, the user can request the construction of a sentence. The frontend then sends the vocabulary set to the ML service, which attaches rules for querying the LLM model. The LLM constructs a sentence and the message travels back through all modules until it reaches the frontend, where it is presented to the user. Finally, this message can be forwarded to any video-calling service.

To sum up, this section outlines a system for LGP interpretation, covering video acquisition, data engineering for building an LGP knowledge pool, ML architectures for sign-by-sign training and inference, a sign tokenization-oriented interaction tool, and the integration of LLM for constructing sentences from inferred words and terms. The next section will delve in the implementation details.

## 4. LGP-To-Text System Implementation

This section provides details regarding the implementation of the LGP-to-Text proposed in this paper, according to the specification above.

### 4.1. Web-Based Tool for Raw Data Collection

The collection of examples of LGP plays a vital role in training and refining models designed for LGP interpretation and generation. This undertaking involves the intricacies of LGP’s gestures, movements, and linguistic nuances. To address this challenge, an initiative was taken to develop a frontend application using Vue.js (Vue.Js—The Progressive JavaScript Framework|Vue.Js, available in https://vuejs.org/, accessed on 14 September 2023), operating within a Node.js (Node.js, available in https://nodejs.org/en, accessed on 14 September 2023) Docker container. The primary objective of this application is to provide an online platform for LGP experts to record videos featuring LGP examples. To operate effectively, this platform incorporates the MediaPipe (MediaPipe|Google for Developers, available in https://developers.google.com/mediapipe, accessed on 14 September 2023) library, which facilitates the detection of contributors’ pose skeletons and provides visual feedback to confirm the successful acquisition of key points.

**Figure 6:**
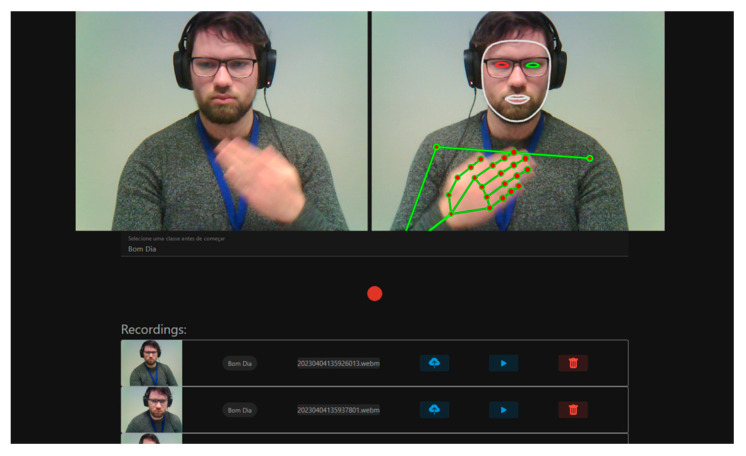
Screenshot of the interface of the gesture video recording application. The video stream on the right-side overlaps with visual elements representing the recognized skeletal key-points (red dots) and the corresponding connections between them (green lines).

In terms of visual/interactive elements, as depicted in Figure 6, this application displays the unprocessed camera feed alongside the MediaPipe-based skeletal and facial detection view. Below, there is a drop-down menu populated with classes (signs) that require recording, prioritized based on the number of examples (videos) available. Users can select a class from the drop-down and initiate recording by performing the corresponding sign. The application waits for the detection of key points corresponding to hand landmarks, triggering the start of the recording. During this process, if hand detection ceases, a predefined timeout period begins. If there is no subsequent hand detection within this period, the recording is considered concluded. The resulting video is stored in the web browser cache and added to a list on the application’s interface. Within this list, users have the option to review, delete, and upload the recorded video to the server.

To complement this platform, a process was defined to allow contributors access to more than one means of recording sign examples, using a shared Google Drive folder created for this purpose. However, this strategy has a few drawbacks: (a) it does not provide immediate insight into whether the gestures were recorded to capture qualitative information regarding contributors’ landmark recognition; (b) it lacks guidelines to ensure proper recording, unlike the previous platform, which starts and stops video acquisition based on hand detection; (c) it does not offer an easy and intuitive way to review videos from a single recording session; and (d) it does not implement features for data security and responsibilities, as anyone with the shared folder link can upload, move and remove all the videos inside. Nevertheless, many contributors preferred the Google Drive option.

### 4.2. Data Engineering toward the Construction of ML-Compliant Datasets

After successfully acquiring all fifty LGP signs, each with 33 examples (as detailed in Table 1 and Table 2), the subsequent steps involved a series of data analysis, augmentation, and structuring processes. To achieve this, various Python 3.8 (Python Release Python 3.8.0|Python.org, available in https://www.python.org/downloads/release/python-380/, accessed on 14 September 2023) scripts were developed, making use of essential libraries such as OpenCV (OpenCV-Open Computer Vision Library, available in https://opencv.org/, accessed on 14 September 2023), Numpy (NumPy, available in https://numpy.org/, accessed on 14 September 2023), and Tensorflow/Keras (TensorFlow, available in https://www.tensorflow.org/, accessed on 14 September 2023). Additionally, the operations related to reading and saving structured comma-separated value (CSV) files in this subsection are executed via Pandas library (Pandas-Python Data Analysis Library, available in https://pandas.pydata.org/, accessed on 14 September 2023).

#### 4.2.1. Step 1: Splitting Data into Train/Validation and Test Subsets

The initial step in managing the acquired LGP sign videos involves their categorization into two distinct subsets: the train/validation subset and the test subset. The train/validation subset serves as the primary dataset used to train the inference models, allowing them to learn the distinctive patterns characterizing each LGP gesture. In contrast, the test set is reserved for evaluating models’ performance on previously unseen data. To ensure the effectiveness of this evaluation, it was essential to establish certain criteria for the testing videos. Specifically, each test video needed to contain a substantial number of landmark-based points sets, with a minimum requirement of 10 sets. To meet this requirement, the MediaPipe library was integrated into the process, which enabled the analysis of each testing video to collect landmark-based points from individual frames. Only frames in which at least one of the hands is detected are considered for this purpose. After analyzing the videos, if the sets of points fell short of the required 10, the video is replaced by another from the train/validation subset and subject to the same assessment. The ultimate objective was to have a minimum of five valid videos for testing the models associated with each LGP sign.

#### 4.2.2. Step 2: Video-Based Data Augmentation

After appropriately dividing the data, the process of augmenting train/validation videos is initiated by implementing shear transformations applied to individual frames. This augmentation process involves extracting all frames from each video in the raw data folder and subjecting them to shear-based distortions. The transformed frames are then reassembled into a new video file, which is saved alongside the original source. Shear transformations are applied by default within a range of −16° and +16°, resorting to Keras’s ImageDataGenerator (tf.keras.preprocessing.image.ImageDataGenerator|TensorFlow v2.14.0, available in https://www.tensorflow.org/api_docs/python/tf/keras/preprocessing/image/ImageDataGenerator, accessed on 14 September 2023). Table 3 confronts original vs. transformed frames.

#### 4.2.3. Step 3: Landmark-Based Sequence Points Extraction with Lateral Dominance Balance

Following the data division, another essential process involves the extraction of human landmarks-based points from both the original and augmented videos. This extraction process relies on the MediaPipe library to identify the *x,y* coordinates of 88 face, hands, arms, and torso landmarks, with a particular focus on the following groups and their respective components:

- Pose landmarks: including right eye, left eye, right shoulder, left shoulder, right elbow, and left elbow;
- Left/right hand landmarks: encompassing various parts, such as the wrist, thumb carpometacarpal joint, thumb metacarpophalangeal joint, thumb interphalangeal joint, thumb tip, index finger metacarpophalangeal joint, index finger proximal interphalangeal joint, index finger distal interphalangeal joint, index finger tip, and corresponding components for the middle, ring and pinky fingers;
- Face landmarks: covering a total of 66 points situated around the upper and lower lips.

An analysis of the frames of each video within a specific sign class folder is involved in the process that resorts to MediaPipe to extract the sequence of positions of the designated landmarks, ensuring that contributors’ motion features are captured. This procedure also includes an additional data augmentation operation that entails horizontally flipping frames to mitigate issues related to lateral dominance (Table 4). Therefore, extraction occurs in both the original video and a corresponding mirrored version. However, if, for any reason, 10 or more sets of 88 points cannot be retrieved by MediaPipe in at least one video within the normal/flipped pair, both videos are discarded to prevent contributing to lateral dominance bias. On the contrary, if the necessary points can be extracted, they are locally saved as independent entries in a CSV file. This file is stored in the same folder as the sign class being processed and contains specific fields for each point set, including a unique subject identification (ID), frame number, 176 *x,y* values representing the landmark positions, and the associated sign label.

#### 4.2.4. Step 4: Point-Based Data Augmentation, Normalization, and Dataset Consolidation

This subsection covers the additional steps required for data preparation, culminating in the creation of a well-structured dataset. These steps encompass point-based data augmentation, implemented in Python, utilizing the previously introduced RRS and SBI techniques. These techniques leverage mathematical libraries such as Numpy to achieve dataset balancing, enlargement, and controlled randomness to enhance diversification. Another critical aspect involves data dimension normalization, aimed at selecting uniformly spaced intermediate points from sign-related data chunks of varying sizes. This normalization process ensures the data’s consistency and readiness for analysis. Finally, all the prepared data are consolidated into a single, comprehensive CSV file. This file contains essential information, including details about the subjects (contributors), labels (signs), and the filtered landmark-based points sourced from both the original records and the augmentation elements.

### 4.3. LSTM Models Training and Deployment

**Figure 7:**
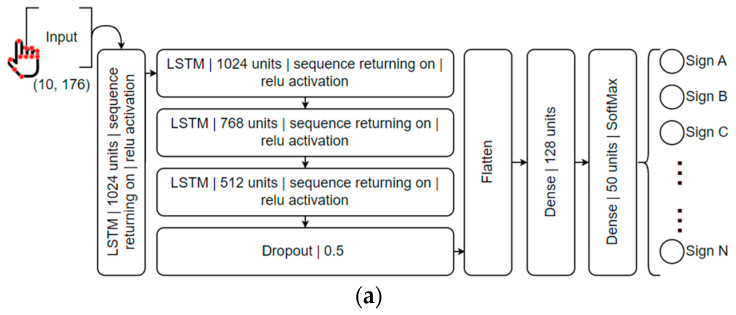

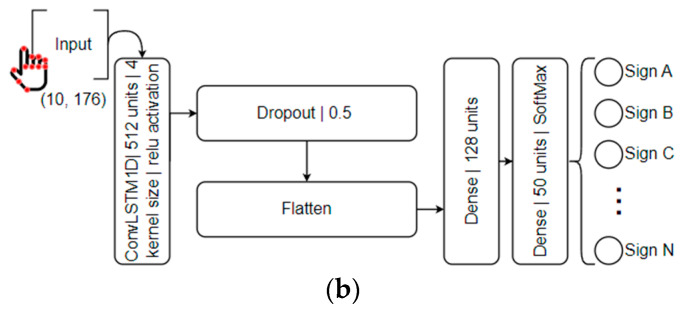
LSTM architectures set up for landmarks-based LGP interpretation. While (**a**) depicts an architecture composed of three simple LSTM nodes, (**b**) shows an architecture with a single layer combining 1D Convolutions and LSTM.

The training of machine learning models was conducted using the TensorFlow/Keras framework. Initially, the CSV containing the tabular dataset is loaded. The dataset is then divided into training and validation subsets through a random split, with a fixed seed to ensure consistent division for the sake of experimental reproducibility. In this work, two LSTM-based structures were used for mapping LGP gestures and motion dynamics, as shown in Figure 7. One structure consisted of three LSTM layers, while the other combined a single 1D Convolutional layer with LSTM. Both accepted an input of 10 sequences, each comprising 176 values representing the positions of landmarks on the torso, arms, hands, and face. For the output, a Dense layer with 50 units was used to match the number of recognizable signs.

Monitoring and optimizing the training of the models are vital aspects of model development. To facilitate this, three essential callbacks were implemented:

- Early stopping: it halts the training process after a specified number of consecutive epochs without significant learning improvements. In this work, the threshold—also known as patience—was set to 30 epochs, the loss associated with validation data was monitored, and a minimum fluctuation of 1 × 10^−4^ was required to consider further learning.
- Model checkpoint: this callback saves models after each training epoch, with a designated format name, allowing for easy tracking of model progressed. Again, the variable to monitor the learning status is the loss associated with validation. It also includes a flag to save models that exhibited improved learning.
- Graphical monitor—TensorBoard (TensorBoard|TensorFlow, available in https://www.tensorflow.org/tensorboard, accessed on 14 September 2023): this tool logs essential training progress, including training accuracy, training loss, validation accuracy, and validation loss, among other metrics. It provides valuable insights and can be accessed through a web-based application.

Consistent training parameters were maintained across all sessions for sign learning. The initial training rate was set to 1 × 10^−4^. The number of training epochs was fixed at 1000, and the batch size used was 128. The Adam optimizer was selected to regulate the learning rate during training. All ML activities were performed on a computer with the following specifications:

- Processor: 11th Gen Intel® Core™ i7-11800H @ 2.30GHz (Intel Co., Santa Clara, CA, USA);
- Random Access Memory (RAM): 32GB @ 2933MHz SODIMM (Corsair Gaming, Inc., Milpitas, CA, USA);
- Graphic Card: Nvidia® GeForce RTX 3080 (laptop edition), 16.0GB GDDR6 RAM (Nvidia Co., Santa Clara, CA, USA);
- Storage: 1TB, 3500MB/R, 3300MB/W (Samsung Electronics Co., Ltd., Suwon, Republic of Korea);
- Operative System: Windows 10 Home 64 Bit (Microsoft Co., Redmond, WA, USA).

### 4.4. Sentence Construction Powered by Chat-GPT

Up to this point, the proposed sign interpretation solution can only recognize LGP, producing individual words/terms (tokens) as its classification output. However, the implementation requires an approach to construct sentences with improved grammatical, syntactical, and morphological structure using these generated tokens. This transformation is aimed at facilitating more coherent communication with potential recipients on the other end of the conversation spectrum. To address this requirement, the utilization of emerging LLM-based tools with NLP and NLG capabilities was considered. A prominent example of such a tool is ChatGPT [14], renowned for its multifaceted application as an AI-powered chat-bot. ChatGPT is proficient at crafting context-aware textual content and can be tailored to follow user-specified guidelines or instructions, thus conditioning the underlying LLM to generate outputs that align with specific criteria. Available through both a web-based chatroom and a REST-based API, ChatGPT showcases remarkable versatility, making it well-suited for enhancing sentence construction within the proposed LGP recognition solution. By incorporating the advanced capabilities of ChatGPT API, the proposed LGP platform gains the functionality to create sentences that not only adhere to linguistic conventions but also align with the specialized needs of SLR. Building upon the ChatGPT API, a set of dynamic and static rules were defined to query ChatGPT3.5 LLM:

- Dynamic rules: “To generate a concise sentence considering the following words/terms and punctuation from the given set S_tokens_”, where S_tokens_ represents a sequence of words/terms, optionally ended by a punctuation mark, more specifically, a “.” or a “?”.
- Static rules: (a) “To ignore repeated words/terms”; (b) “To restrain, as much as possible, to the tokens that there are in the given set, avoiding to add more”; (c) “To consider the present indicative by default;” (d) “If a personal pronoun is not indicated, to conjugate the verbs in the first person singular”; (e) “To perform only minimal transformations to the words¨, with the goal of ensuring grammatical correctness”; (f) “To perform spelling corrections, whenever necessary”; (g) “To correct gender and number agreement inconsistencies”; and, finally h) “To interpret numbers as quantifiers”.

This rule set was initially tested and refined within the chatroom provided for direct interaction with ChatGPT’s LLM. Subsequently, an API-based bridge was established for the proposed LGP recognition platform to integrate this NLG tool, which returns ready-to-use and straightforward answers without the need for additional parsing.

### 4.5. Web-Based Central Service Layer

To bring all the components of the proposed LGP system together within a harmonized response/request communication context, a prototyped web-service was developed with the FastAPI (FastAPI, available in https://fastapi.tiangolo.com/, accessed on 14 September 2023) framework on a Uvicorn-enabled Docker container (Uvicorn—Docker FastAPI projects 0.0.2 documentation, available in https://docker-fastapi-projects.readthedocs.io/en/latest/uvicorn.html, accessed on 14 September 2023). This framework, allows the definition of Web API logic within a single script, making it easy to create consumable endpoints. With a common Python-based environment to bridge external requests with Tensorflow and Keras libraries and their functionalities, as well as with third-party REST APIs, the integration of features, such as deployed models for sign inference and communication with ChatGPT extended sentence-building capabilities, occurs smoothly and seamlessly. After the server initialization and the loading of LGP inference models, a series of endpoints are made accessible, as listed below:

- *config*—provides an external service consumer with the current prediction configurations, offering information about the available vocabulary sets and LGP models to use, as well as the expected landmarks to monitor, assuming MediaPipe data formats;
- *vocab_selector*—allows the switching between existing vocabulary and model combinations (compliant with the data provided by *config* endpoint);
- *mp_estimator*—returns word/expression predictions upon a provided list of coordinates sent by an external service consumer;
- *sentence_GPT*—when provided with a list of words from an external service consumer, this endpoint generates a prompt for ChatGPT API, yielding a simple but semantically and grammatically consistent sentence integrating the referred list of words.

The multiple references to “external service consumer” should be interpreted as an independent application layer that needs to communicate with the LGP core services to obtain inferences. An example of an external consumer will be given in the following subsection, which presents a frontend tool that resorts to a video feed from the camera of a given device in use to obtain user landmarks’ positions and perform requests to the inference capabilities gathered by this central web-service layer.

### 4.6. Deaf-Side Frontend Experimental Tool

**Figure 8:**
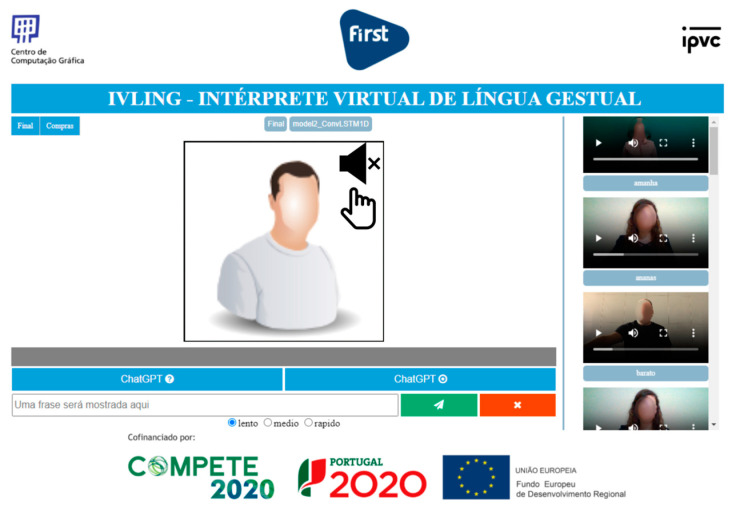
Layout of the experimental frontend for interoperating with the proposed LGP recognition platform.

To demonstrate the interoperability with the central web-service layer and its inference capabilities, an independent Javascript-based frontend application was experimentally developed. As shown in Figure 8, this application features a minimalistic design and interaction. It seamlessly integrates the Javascript version of MediaPipe to facilitate user landmark detection through camera feed processing and connects with endpoints specified in the previously presented service layer, including *config*, *vocab_selector*, *mp_estimator*, and *sentence_GPT*.

In terms of the frontend’s functionalities, users are initially required to wait for MediaPipe to load, resulting in the camera feed becoming available to capture user actions. Subsequently, users can choose their preferred speed from options such as “slow” (by default), “medium”, and “fast” based on their experience and dexterity. When the camera detects the user’s hands, landmark-based points start to be collected into an array, while a progress bar begins filling up according to the selected speed. This process encourages users to synchronize their actions with the visual element. Once the bar is fully filled, it signifies the completion of the LGP term. The collected data are then forwarded to the prediction model through a dedicated endpoint (as previously described in *mp_estimator*), and the most relevant term from the learned vocabulary is returned. Upon accumulating a satisfactory list of terms, users can request a simple yet properly articulated sentence through a dedicated endpoint established for this purpose (as described in *sentence_GPT*).

The following section will present the primary tests and their corresponding results, covering various aspects of the LGP platform, such as landmark-based points extraction features (before dataset consolidation), LGP inference capabilities (after model training), and the ability to construct sentences, among others.

## 5. Tests and Results

Several tests were conducted to assess the proposed LGP interpretation solution, focusing on the distinct perspectives that are worthy of focus, from dataset preparation to AI-related features for language sign-based word/term recognition and sentence generation.

### 5.1. Landmark-Based Points Extraction

**Figure 9:**
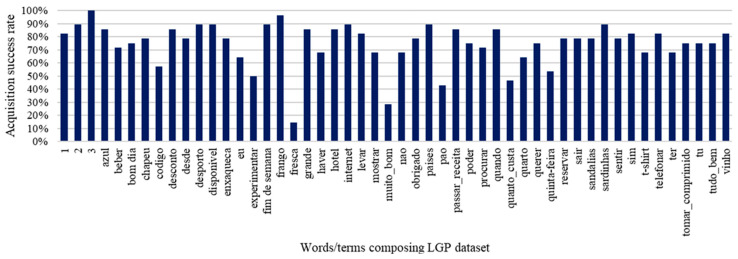
Overview of the percentage of the usable train/validation signs, after video-based data extraction process. Terms *fresca*, *muito_bom*, *pao*, and *quanto_custa* were the only ones below 50%. Most of the signs, more specifically, 36 of them, were above the rate of 70%.

After executing the process for feature extraction based on human landmarks detected from LGP videos provided by contributors, an overall success rate of 74.6% was attained across the examples recorded for each of the 50 signs. The plot in Figure 9 provides an overview of the extraction results, wherein poor percentages can be noticed for the words/terms “fresh” (*fresca*), “very good” (*muito_bom*), “bread” (*pao*), and “How much does it costs” (*quanto_custa*), in contrast with “2” (*dois*), “3” (*três*), “sports” (*desporto*), “available” (*disponível*), “weekend” (*fim de semana*), “chicken” (*frango*), Internet (the same in Portuguese), ”countries” (*países*), and “sardines” (*sardinhas*).

Therefore, significant class balancing is needed, besides regular data augmentation. For the results yet to be shown with direct regard to dataset usage, this need is an aspect to consider, to mitigate the production of largely biased and, therefore, unusable models.

### 5.2. LGP Models Parameters

To better understand word/term inference models’ performances presented subsequently, inspecting models’ trainable parameters can be insightful. Hence, Table 5 presents the data regarding not only that information, but also the average time spent per training epoch, for both of the LSTM-based architectures used to produce LGP inference models, more specifically, one combining pure LSTM layers with different configurations at the filter level (SimpleLSTM) and, another, structurally simpler, that has a top layer combining LSTM with convolutional features (ConvLSTM). While, in the former, 13.221.554 trainable parameters were detected, in the latter, that number reached 15.548.850.

Notwithstanding, nothing can be concluded regarding their inference capabilities, comparatively to each other. To address this specific topic, the next subsection presents the results of empirical tests conducted with various models derived from the previously identified LSTM architectures. These tests involved varying training conditions, mainly associated with specific operations aimed at optimizing the dataset for the achievement of enhanced inference models.

### 5.3. LGP Terms Inference Accuracy

In this subsection, a series of tests conducted with models trained with both presented architectures (SimpleLSTM and ConvLSTM) are detailed, and the respective results are shown. To start, the simpler architecture, i.e., SimpleLSTM, was submitted to a series of tests/assessments involving several variations of the LGP dataset, as shown in Table 6. For comparison purposes, the pair of datasets adjusted with the dominant side calibration induced by the horizontal flips (HF) and then, combining the former transformation with shear operations (SO) were not balanced/augmented. For the remaining datasets involving RRS, SBI, or both, augmentations aiming at balancing examples and improving diversity were carried out. To establish a ceiling for augmentations affecting the component of the dataset used for training, Equation (2) was defined:

(2) $$Limit_{class}=CN\times DE\times NP+CN$$

where:

$Limit_{class}$ is the limit for data augmentation, per class;
CN represents the number of elements, per class (in unbalanced data, the class with the higher number of examples is the reference);
DE stands for the number of dataset augmentation transformers, besides the use of original data;
NP is the number of passages specified for applying dataset augmentation transformers.

Therefore, the following set of specific values was considered to constrain the data growth induced by the aforementioned augmentations:

- CN = 28 signs × 2 (from HF) × 2 (from SO) = 168;
- DE = 1, even for the dataset involving inline RRS/SBI augmentation, wherein both RRS and SBI are carried out combined in the same operational flow for data transformation;
- NP = 5, regarding the number of passages to apply DE.

Hence, 168 × 1 × 5 + 168 = 1008 examples, including original and augmented data. As it can be observed from Table 6, the dataset variants involving RRS transformations were the ones that contributed to obtaining the top-2 SimpleLSTM versions, when testing the resulting models against unseen data. In the next stage, these RRS-based variants were chosen to test ConvLSTM, instead of utilizing all the dataset versions, therefore avoiding running unnecessary training sessions. The respective results can be found in Table 7.

In direct comparison with SimpleLSTM, more specifically, considering homologous datasets, ConvLSTM achieved better results. Moreover, one can observe that training with datasets transformed resorting to HF + SO + RRS led to more accurate models than using the RRS/SBI combination in both architectures.

**Figure 10:**
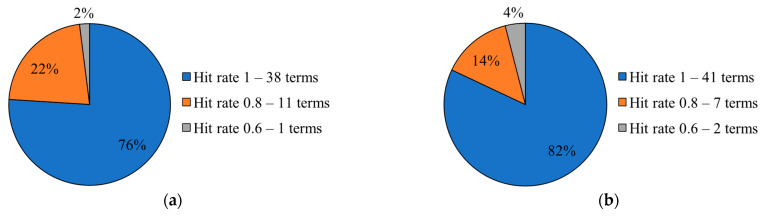
Hit rate comparison for SimpleLSTM vs. ConvLSTM, considering the dataset augmented with HF, SO, and RRS: (**a**) SimpleLSTM hit rate plot; and (**b**) ConvLSTM hit rate plot.

Focusing on the top-1 models of SimpleLSTM and ConvLSTM architectures, the hit rates for the 50 collected LGP signs, i.e., the percentage of correct observations per word/term after submitting the respective unseen data (videos) to inference, were measured. To that end, the inference results were organized by grouping the words/terms into three observed accuracy scales, more specifically, 1, 0.8, and 0.6 (Figure 10). LSTMConv model had more words/terms predicted seamlessly (i.e., 82% of the signs), but, also, a higher hit rate in the most lossy group (i.e., 4% of the signs predicted with only 60% of accuracy).

Regarding SimpleLSTM, the words/terms that were seamlessly classified are *2*, *3*, *azul*, *chapéu*, *desconto*, *desde*, *desporto*, *disponível*, *enxaqueca*, *eu*, *fim de semana*, *frango*, *fresca*, *grande*, *hotel*, *internet*, *levar*, *mostrar*, *obrigado*, *países*, *pão*, *passar*_*receita*, *procurar*, *quando*, *quanto_custa*, *quarto*, *querer*, *quinta-feira*, *reservar*, *sair*, *sandálias*, *sardinhas*, *sentir*, *t-shirt*, *telefonar*, *tomar_comprimido*, *tudo_bem*, and *vinho*. The ones that attained 80% of accuracy are 1, *beber*, *bom dia*, *código*, *haver*, *muito_bom*, *não*, *poder*, *sim*, *ter*, and *tu*. With 60% of match, a single word remained: *experimentar*. As for the ConvLSTM, *1*, *2*, *3*, *azul*, *beber*, *chapéu*, *desconto*, *desde*, *desporto*, *disponível*, *enxaqueca*, *eu*, *fim de semana*, *frango*, *fresca*, *grande*, *haver*, *hotel*, *internet*, *levar*, *mostrar*, *obrigado*, *países*, *passar_receita*, *poder*, *procurar*, *quando*, *quanto*_*custa*, *quarto*, *querer*, *quinta*-*feira*, *reservar*, *sair*, *sandálias*, *sardinhas*, *sentir, t-shirt*, *telefonar*, *tomar*_*comprimido*, *tudo*_*bem*, and *vinho*, were the words/terms with full matching against the testing set. Having 80% of correspondence, were identified *bom dia*, *código*, *muito*_*bom*, *não*, *sim*, *ter*, and *tu*, while the ones with worst the matching rate (60%) were *experimentar*, and *pão*.

Next, the NLG capacities of ChatGPT for the articulation of sentences based on a set of prespecified conditions and tokens will be carried out, in alignment with the requirements of the proposed LGP platform.

### 5.4. Tests to Chat-GPT Restriction Rules for Conditioned Sentence Generation

After acquiring the terms/words (tokens) from LSTM models, the construction of well-structured and contextualized sentences can be carried out by using an LLM, more specifically, the one that supports ChatGPT. To assess its NLG capabilities for the intended task, a testing environment was defined, considering the following steps: (i) selection of three conversation contexts, commerce, tourism, and health care; (ii) definition of declarative or interrogative sentences for each defined context, containing only the words/terms of the lexical field supported by the previously presented LSTM models, also used as ground truth (i.e., expected LLM outcome); (iii) obtention of ChatGPT-generated text, by querying it using the tokens defined for each previously defined sentence along with a preestablished set of conditioning rules, identified in Section 4.4; and (iv) semantic comparison between the expected sentences and the responses given by ChatGPT. For assessing the expected sentences against the output, the model kit of the universal sentence encoder [29] was used. It can make semantic-aware mapping of sentences into embeddings that correspond to vectors of continuous values, numerically comparable. The recommended metric to perform these comparisons and measure similarities, accordingly with [29], must first take into account the computation of *cosine* similarity of the two sentence embeddings, and, then, the *arccos* to convert the *cosine* similarity into an angular distance, as shown in Equation (3). Semantic correlation results can be found in Table 8.

(3) $$sim\;\left(u,v\right)=1-\frac{arccos\left(\frac{u.v}{\left\|u\|\|v\right\|}\right)}{\pi }\;$$

where:

*u* and *v* represent the embeddings of the sentences involved in the assessment.

**Table 8:** Evaluation of ChatGPT’s NLG capacities, according to the requirements of the proposed LGP recognition platform. The universal sentence encoder [29] was used to retrieve semantic correlations between expected sentences and ChatGPT responses (“semantic similarity” column), in a scale from 0 to 1, wherein 1 means a perfect match.

Context	LGP Gloss	Expected Sentence	ChatGPT Sentence	Semantic Similarity
Commerce	*FRANGO QUERER*	*Quero frango.*	*Eu quero frango.*	0.79
	*QUANTO.CUSTA?*	*Quanto custa?*	*Quanto custa?*	1.00
	*PÃO TRÊS LEVAR*	*Levo três pães*	*Levo três pães.*	1.00
	*SARDINHAS HAVER?*	*Há sardinhas?*	*Há sardinhas?*	1.00
	*SARDINHA DOIS QUERER*	*Quero dois sardinhas?*	*Eu quero dois sardinhas.*	0.67
	*T-SHIRT DESPORTO HAVER?*	*Há t-shirts de desporto?*	*Há uma T-shirt de desporto?*	0.75
	*AZUL GRANDE TER?*	*Tu tens azuis, grandes (tamanho)?*	*Tu terás um grande azul?*	0.37
Tourism	*QUARTO DISPONÍVEL QUINTA.FEIRA TU TER?*	*Tu tens quarto disponível para quinta-feira?*	*Tu terás um quarto disponível na quinta-feira?*	0.59
	*RESERVAR QUERER*	*Quero reservar.*	*Quero reservar.*	1.00
	*HOTEL INTERNET TER?*	*O hotel tem Internet?*	*Ter internet no hotel?*	0.74
	*CÓDIGO TU TER?*	*Tu tens o código?*	*Tu tens código?*	0.91
	*FIM.DE.SEMANA TU DISPONÍVEL?*	*Tu estás disponível no fim de semana?*	*Tu tens disponível o fim de semana?*	0.80
	*PAÍS (TAMBÉM) HOTEL TU PROCURAR*	*Procuras país e hotel?*	*Tu procuras hotel no país?*	0.62
	*SAIR QUANDO?*	*Quando é para sair?*	*Quando saio?*	0.70
	*T-SHIRT CHAPÉU SANDÁLIAS TU LEVAR*	*Tu leva chapéu, t-shirt e sandálias.*	*Eu levo a T-shirt, o chapéu e as sandálias.*	0.59
	*VINHO SARDINHAS FRESCAS EXPERIMENTAR QUERER*	*Quero experimentar vinho e sardinhas frescas.*	*Quero experimentar vinho e sardinhas frescas.*	1.00
Heath care	*ENXAQUECA SENTIR QUINTA-FEIRA DESDE*	*Sinto enxaqueca desde quinta-feira.*	*Sinto enxaqueca desde quinta-feira.*	1.00
	*TU PASSAR.RECEITA?*	*Tu passas receita (médica)?*	*Tu passas a receita?*	0.91
	*VINHO BEBER PODER?*	*Posso beber vinho?*	*Posso beber vinho?*	1.00
Average Similarity:				0.81

### 5.5. Tests with an End-User

As previously explained, the tokenization of terms/words is accomplished through an interaction technique involving synchronization with a progress bar-like visual feedback component while performing a sign. Therefore, a series of pilot tests to evaluate participants’ intuition regarding the use of such an interaction-based tokenizing approach was carried out. A total of 12 participants with academic degrees in diverse backgrounds, including Civil and Electrotechnical Engineering, Climatology, Computer Sciences, Forest Planning and Management, and Geographic Information Systems (GIS), were recruited from a university context. Out of the participants, seven were male adults, while the rest were female adults.

**Figure 11:**
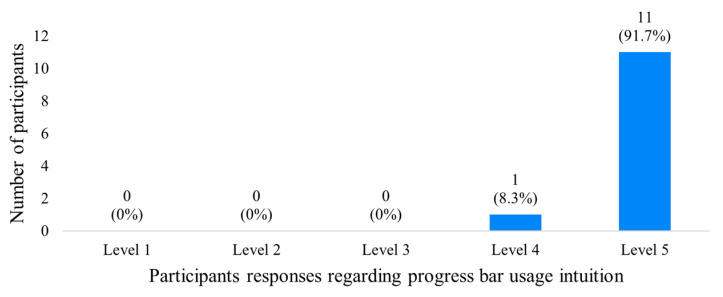
Participants’ feedback regarding the intuition provided by the proposed progress bar-based interaction technique for performing synchronized signs for effective tokenization.

In terms of procedures, the tests were conducted in two main stages: pretraining and effective evaluation. During the pretraining stage, participants familiarized themselves with a frontend layout developed to interface with the proposed LGP platform. They were also introduced to some signs and the synchronization progress bar under evaluation. In this stage, participants were instructed to position themselves in front of the camera, ensuring they were at the center of the frame (verifiable by a camera-related image rendering). They were informed that gesture capture only occurred when at least one of their hands was within the camera’s viewport. Additionally, the progress bar feature was explained, and participants were given access to a couple of sign videos, including *pão* (bread) and *querer* (to want), for analysis and practice. This exercise aimed not only to enhance participants’ understanding of the reception of inferred terms/words and graphical user interface (GUI) updates but also to familiarize them with the dynamics of the progress bar. After reaching a level of comfort to move on to the next stage, the participants were invited to, autonomously perform signs in a certain order, from a new set of examples—*chapéu* (hat), *experimentar* (to try), and *querer* (to want)—which were provided for being freely consulted. During this second stage, synchronization and inference errors were registered. Finally, they were asked to fill out a very small and pragmatic questionnaire that, besides demographic data, and consent confirmation, had a single question, regarding their felling about the intuition provided by the proposed interaction technique for performing synchronized signs and, therefore, achieving effective tokenization, by resorting to a Likert scale of five levels, wherein the higher one corresponds to plain satisfaction. Participants’ feedback is consolidated in the plot of Figure 11.

As observable, there is a clear tendency for the top levels of intuition in the usage of the proposed progress bar-based interaction. Of the 12 participants, 11 reported plain intuition feeling, while only one responded with the fourth level. In terms of the errors made during the effective evaluation stage, the following data were registered:

- Total of synchronization errors: 1 (0.08 of mean);
- Total of inference errors: 14 (1.27 of mean).

Overall, the proposed interaction technique seems to be suitable for sign tokenization. As for the inference errors, they can be explained by the lack of experience in LGP that characterized the sample. Moreover, the several observational tests carried out with a Deaf person of APS corroborate the same conclusions that were drawn in the tests made with these 12 participants, since that user did not present any difficulties in interacting with the progress bar while performing signs. Although a pre-operational familiarization stage seems to be recommendable before effective utilization.

### 5.6. Integrated Frontend Layer Functional Testing

**Figure 12:**
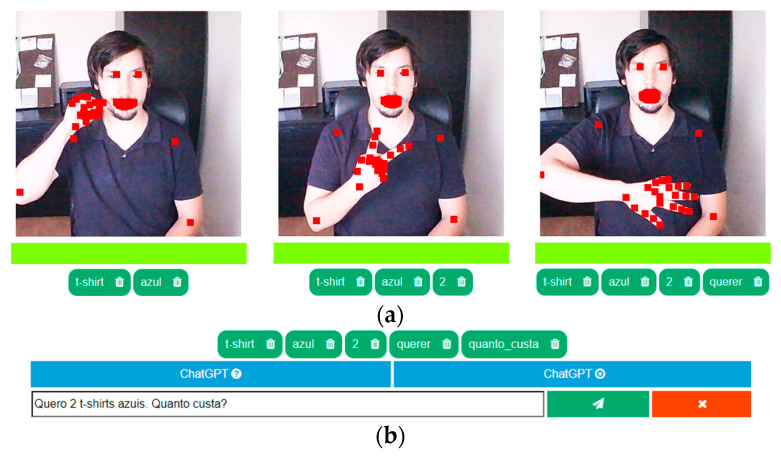
Layout of the frontend used to perform functional tests on the proposed LGP platform. In (**a**) there is a user gradually performing signs that are converted in words/terms by inference of the ConvLSTM model incorporated in the proposed platform; in (**b**), a well-structured sentence can be observed, which resulted from submitting the inferred list of terms/words to ChatGPT’s API service, via proposed platform.

A few tests were carried out to assess the interoperability potential of the proposed LGP platform, along with the interactive and visual features provided through an individual frontend that was developed and incorporated to consume endpoints on the platform’s side, as previously detailed. The tests consisted of going through the whole process for LGP interpretation until meaningful sentence production from the user’s perspective. Therefore, the first step is to perform signs, term-by-term, to the frontend, allowing the camera to capture the user while the respective anatomic landmarks are processed via MediaPipe and collected for an array of point sets, in a synchronous manner with the progress bar. Each sign’s data flow to the remote LGP platform that responds with a matching word/term. After gathering a comfortable vocabulary set, the user requests the production of a sentence to the NLG service, using the LGP platform, that consults ChatGPT API using not only that group of terms/words but also a prespecified set of conditioning rules. Finally, the sentence is shaped up and returned to the user, closing the process. Figure 12 depicts one of the tests made with the words “*t-shirt*”, “*azul*” (blue), “*2*”, “*querer*” (to want), “*quanto_custa*” (how much does it costs?).

In terms of system efficiency, the landmark-based points set have a smooth recognition rate, between 20–30 frames per second (FPS) while working with MediaPipe for the recognition of a single skeleton, using a laptop with the specifications identified in Section 4, plugged into the electrical outlet, i.e., not relying on the computational profile when solely powered by a battery. In smartphones, depending on the computational capabilities, a drop in FPS is likely to be experienced. As for the LGP platform’s LSTM-based service responsible for the conversion of signs into text, usually, times of response take less than 500 milliseconds, between the frontend request and the reception of the result. Moreover, the sentence generation functionality, through ChatGPT, performed reasonably well with a user-based on-demand request strategy (by button-clicking interaction), with responses taking less than 1s, in general. Although, with the implementation of an intensive generation functionality, responsive to changes in the word list at the frontend side, a hang problem with the endpoint was identified, probably, due to OpenAI servers overloading, which results in considerable execution locks, of several minutes, sometimes. Therefore, it is advisable to leave the responsibility of querying ChatGPT to the user instead of relying on the system every time a change in the list of words occurs.

After the presentation of the main results obtained from the several tests made to the LGP platform, a discussion will take place in the following section.

## 6. Discussion

There are two points of discussion aligned for this section: the first one provides a global appreciation of the obtained results in this work, and the second one includes a comparative analysis regarding the solution proposals reported in the literature.

For starters, landmarks extraction from video examples will be addressed, wherein a mean rate of 74.6% was achieved. The issues associated with losses in more than 25% of the signs provided by contributors may have several explanations: (a) the length of the videos, which in some cases was too short to allow the capturing of sufficient point sets; (b) the poor capturing conditions that were noticed in many of the recorded videos that may have hampered the detection of landmarks; and (c) limitations associated to MediaPipe itself, more specifically, in situations wherein more demanding poses are verified (for instance, if the hand assumes a position relative to the camera viewport in such a way that anatomical points may look as if overlapped, causing the detector to malfunction). Despite these more challenging occurrences, most of the examples were able to consistently provide valuable data, in both quality and quantity, for being used in the steps subsequent to point extraction.

Regarding models’ production for individual vocabulary inference, ConvLSTM could retrieve more trainable parameters than SimpleLSTM, in spite of being structurally simpler. Underlying this observation may be the convolutional layer associated with LSTM architectures, which seems to have promoted a deeper dataset inspection. On the other hand, ConvLSTM was able to outperform SimpleLSTM in terms of accuracy (95.6% vs. 94.8%), but with significant implications for training time, which was, on average, 17.5 times higher. Hence, trainable parameters and training times seem to have a correlation with accuracy, which, however, cannot be confirmed without performing more tests. Moreover, among the datasets engineering strategies that contributed more to obtaining the inference models with the higher performances were data balancing combined with RRS-based augmentation. The SBI data augmentation permitted to increase in the accuracy of the models built from both adopted LSTM architectures comparatively to the ones that were trained with non-augmented datasets, but it was not as effective as RRS.

In terms of sentence construction, the tests conducted with ChatGPT were successfully completed, yielding promising results that confirm the LLM’s ability to consistently generate valid sentences from sets of LGP words/terms. A key factor contributing significantly to this performance is the predefined set of regulatory specifications (rules) that serve as effective directives for the LLM. Quantitatively, the sentences produced by the LLM matched the expected ones with an accuracy of around 81%. Further refinements to the rules for conditioning ChatGPT outcomes could potentially lead to even higher correlation rates.

The tests made to the tokenization approach based on the proposed progress bar interaction technique showed unanimous agreement among 12 participants, all of whom classified it as highly intuitive. However, some time to allow the user to become accustomed may be advisable before the effective utilization of the graphical interface implementing this strategy. Moreover, while the proposed LGP platform stands as independent from an operative frontend, the responsibility of performing a proper implementation of the interaction dynamics is transferred to the programmers entrusted with coding the intermediary user interface, leading, in turn, to the need of being aware of the intricacies underlying the interoperability requirements.

In regard to the tests conducted to evaluate the interoperability potential of the proposed LGP platform with an external user interface tool developed for that purpose, the results indicated high responsiveness. Terms/words can, indeed, be instantaneously inferred and delivered to the requester, and sentences can be promptly assembled, especially under conditions of non-intensive usage of the ChatGPT API.

Comparatively to other works’ main features, the proposed LGP platform stands out for being non-invasive and for not requiring special motion acquisition devices, oppositely to [4], which resorts to a sensor glove, or to [5], wherein a Microsoft^®^ Kinect is used. Regarding image stream-based recognition accuracies, [6] reached 98% with 10 categories, while, in [7], 100% accuracy was reported during tests with LSA64, by reserving 90% of the data for the training subset. For the Persian dataset, the correspondence rate reported in [8] was 99.5%. While most of these works seem to present higher accuracies compared to the solution proposed in this paper, the differences in the datasets’ overall characteristics must be considered, for a fairer assessment, namely:

- In [6], only 10 classes were regarded in the study—a categorical complexity 80% lesser than the proposed LGP dataset;
- In [7], the reference dataset was LSA64, having 3200 usable examples distributed among 64 classes—28% more classes than those composing the proposed LGP dataset, but, also, around twice of the exemplifying videos, from which 90% was assigned for training subset;
- Finally, in [8], the Persian dataset composed of 100 examples per 100 signs was used–twice of the classes composing the proposed LGP dataset, but 8 times more exemplifying videos, as well.

Others [9], aiming at tackling issues that include sentence segmentation and word alignment, addressed continuous SLR in a dataset composed of 25K videos involving (human) sign language interpreters and managed to achieve 81% sentence recognition matching. With similar goals, the sentence-building approach implemented in the context of the proposed LGP interpretation solution was able to achieve comparable results, more specifically, in the semantic correlation between computer-generated and human-defined expected sentences. This was accomplished through a combination of strategies, including the integration of an interaction-based technique for the tokenization of signs representing words/terms and, also, ChatGPT with advanced grammatical and semantic capabilities, forged to provide human-like text generation. The primary advantage of this approach, compared to [9], seems to lie in its ability to adapt and enhance the interpretation of words/terms for sentence articulation through simply fine-tuning the conditioning rules of ChatGPT, which can be made without the necessity for additional labeling, training sessions, etc.

To conclude the current section, some features that stand out in this work comparatively to others are noteworthy to highlight. Firstly, other proposals in the literature with such a volume of LGP vocabulary dataset, i.e., 50 non-statical signs, corresponding to actual words and terms, and not only numbers or alphabet letters, could not be found. Furthermore, aspects associated with dataset engineering (for example, RRS vs. SBI) that may be valuable for other works as well (for example, human–computer interaction for robotics) are posed and addressed, while perspectives regarding approaches for dataset configurations and sequence-sensitive neural networks (LSTM with and without convolution layers combined) are also provided. Moreover, the proposed LGP platform focuses not only on image stream-based word/term inference but also sentence construction, resorting to novel available LLM approaches, covering the full spectrum of a dialog intention, which is a range that seems to be rarely found in the other literature works. Even scarcer are the considerations related to the design of service-oriented architectures (SOA) within the context of Deaf inclusion, particularly with regard to paving the conditions for streamlining service integration, distribution, and broad-scale adoption.

## 7. Conclusions and Future Work

This paper has presented a comprehensive LGP interpretation system, designed as a service (SOA-oriented), that leverages ML-driven motion analytics approaches for the interpretation of LGP signs extracted from video/camera image streaming. To sustain the ML approaches, a substantial LGP dataset, comprising 50 unique terms, was meticulously curated to facilitate the model training process. However, the acquisition endeavor resulted in a data shortage that had to be properly addressed with strategies for dataset enhancement/augmentation. Therefore, RRS and SBI approaches were implemented and compared. The former demonstrated superior efficacy over the latter, contributing to more accurate and robust inference models, derived from two main LSTM architectures: ConvLSTM and SimpleLSTM. ConvLSTM emerged as the standout performer, surpassing the SimpleLSTM, reaching an accuracy rate of 95.6%. Addressing the tokenization of LGP vocabulary, real-time visual feedback for providing the user a means for monitoring sign-by-sign execution was proposed. This dynamic progress indicator allows users to gauge the remaining time required to complete an LGP expression, enhancing intuition, as corroborated by tests made with participants. Furthermore, the integration of an LLM in the proposed LGP platform, more specifically ChatGPT, showcased its proficiency in constructing coherent and semantically accurate sentences from the terms/words inferred by the LSTM models. The successful integration of ChatGPT adds an extra layer of sophistication to the LGP interpretation system, enabling seamless sentence assembly from individual components. In the end, an independent testing graphical interface developed to bridge with the LGP platform, with the aim of testing all the services implemented, allowed us to conclude that the resulting system has a suitable responsiveness, for both word/terms inference and conditioned sentence construction, with quite acceptable response times.

In the future, there are several avenues to explore for further advancing the proposed LGP interpretation system. Firstly, expanding the LGP vocabulary beyond the initial 50 terms and, also, increasing the number of examples per sign, could significantly enhance the system’s usability and applicability. The inclusion of a more diverse range of terms would offer a more comprehensive interpretation of LGP signs, catering to a broader array of communication needs. Additionally, the exploration of alternative data augmentation techniques remains an intriguing prospect. Experimenting with novel augmentation strategies could potentially address scenarios where scarcity of examples persists, thus bolstering models’ ability to generalize and infer accurate interpretations. Further advancement could be achieved by venturing into alternative neural network architectures, such as Transformers. These architectures have demonstrated remarkable success in various natural language processing tasks and could offer valuable insights into improving the precision and efficiency of LGP interpretation. Moreover, refining the conditioning rules of ChatGPT presents an opportunity to enhance the quality of generated sentences. By fine-tuning ChatGPT’s responses through continuous feedback and iterative training, it is possible to achieve more contextually accurate and linguistically refined outputs, thereby elevating the overall coherence and semantics of the assembled sentences.

## Figures and Tables

**Table 1:** Set of 50 alphabetically sorted Portuguese terms/words to record during the collection campaign. This list includes adverbs, verbs, subjects, definite/indefinite articles, substantives, prepositions, as well as several pronouns (personal/demonstrative/indefinite), among others, and targets shopping, tourism, and medical care contexts.

Portuguese	English (Terms 1–25)	Portuguese	English (Terms 26–50)
*1/um*	one	*não*	No
*2/dois*	two	*obrigado*	thank you
*3/três*	three	*países*	countries
*azul*	blue	*pao*	Bread
*beber*	to drink	*passar (receita)*	to prescribe
*bom dia*	good morning	*poder*	to be able to
*chapéu*	hat	*procurar*	to search
*código*	code	*quando*	when
*desconto*	discount	*quanto custa*	how much does it cost
*desde*	since	*quarto*	room
*desporto*	sports	*querer*	to want
*disponível*	available	*quinta-feira*	Thursday
*enxaqueca*	migraine	*reservar*	to reserve
*eu*	I	*sair*	to leave
*experimentar*	to try	*sandálias*	sandals
*fim de semana*	weekend	*sardinhas*	sardines
*frango*	chicken	*sentir*	to feel
*fresca*	fresh	*sim*	yes
*grande*	large	*t-shirt*	t-shirt
*haver*	to have	*telefonar*	to call
*hotel*	hotel	*ter*	to have
*internet*	internet	*tomar (comprimido)*	to take (a pill)
*levar*	to take	*tu*	you (informal)
*mostrar*	to show	*tudo bem*	okay
*muito bom*	very good	*vinho*	wine

**Table 2:** Summary of the campaign carried out to collect 1650 LGP videos focusing on the context of shopping, tourism, and medical care, resulting in a total of 33 examples for each of the 50 LGP signs.

Total of Examples	Data per Sign				
	Videos	Testing Videos	Train/Validation Videos	Train Percentage	Validation Percentage
1650	33	5	28	~65%	~35%

**Table 3:** Video-based data augmentation, with shear transformation. The top row displays the appearance of the original video frames, while the subsequent rows depict the frames’ appearance after shear transformations at −16° and 16°, respectively.

Transformation State	Frames Aspect
Original	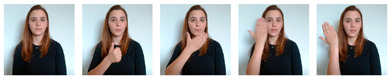
Shear −16°	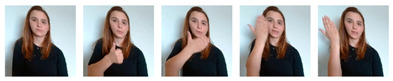
Shear 16°	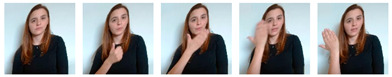

**Table 4:** Video-based data augmentation, with horizontal flipping operations, used to obtain the flipped points from the original video, and, therefore, tackle lateral dominance that may bias the models’ training process.

Transformation State	Frames Aspect
Original	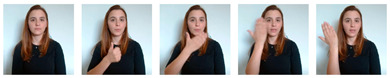
Horizontal flip	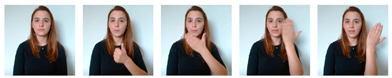

**Table 5:** Data regarding the training parameters found for each LSTM model presented previously: (a) Simple LSTM; and (b) Convolutional LSTM.

Model	Total Parameters	Trainable Parameters	Non-Trainable Parameters
SimpleLSTM	13.221.554	13.221.554	0
ConvLSTM	15.548.850	15.548.850	

**Table 6:** Results of the tests conducted using SimpleLSTM, resorting to several variations of the achieved LGP dataset.

Model	Augmentation Conditions	Augmentation Limit	Accuracy	Loss	Final Epoch
SimpleLSTM	Horizontal flips (HF), not augmented (NA), not balanced.	N/A	80%	1.23	155
	HF + shear operations (SO), not augmented, not balanced.	N/A	87%	0.98	100
	HF + SO + RRS augmentation, balanced.	1008	94.8%	0.47	110
	HF + SO + SBI augmentation, balanced.	1008	92%	0.72	100
	HF + SO + inline RRS/SBI augmentation, balanced.	1008	93.8%	0.444	75

**Table 7:** Results of the tests conducted using ConvLSTM, resorting to RRS-based data augmentations.

Model	Augmentation Conditions	Augmentation Limit	Accuracy	Loss	Final Epoch
ConvLSTM	HF + SO + RRS augmentation, balanced.	1008	95.6%	0.647	85
	HF+ SO + inline RRS/SBI augmentation, balanced.	1008	94.4%	0.455	150

## Data Availability

The data supporting this work is not publicly available due to privacy, ethical, and legal restrictions related to sensitive content (unauthorized sharing of faces) and intellectual property concerns.
